# Older People’s Neighborhood Perceptions Are Related to Social and Emotional Loneliness and Mediated by Social Network Type

**DOI:** 10.1093/geront/gnac087

**Published:** 2022-06-12

**Authors:** Christine Stephens, Hannah Phillips

**Affiliations:** School of Psychology, Massey University, Palmerston North, Aotearoa/New Zealand; School of Psychology, Massey University, Palmerston North, Aotearoa/New Zealand

**Keywords:** Aging, Environment, Health, Housing

## Abstract

**Background and Objectives:**

Loneliness among older people is a public health issue; however, there is very weak support for the efficacy of individually focused interventions. A public health model, which includes the environmental influence on the formation of social networks and protection from loneliness, and theoretical approaches differentiating between social and emotional loneliness, suggest the importance of neighborhoods in preventing loneliness. This approach was used to test the influence of neighborhood factors on loneliness and the mediating role of social networks.

**Research Design and Methods:**

A questionnaire survey of 917 people aged 60–100 years was conducted in one region of Aotearoa/New Zealand to assess loneliness, social network types, social participation, marital status, gender, health, and four aspects of neighborhood perceptions.

**Results:**

Social and emotional loneliness scores were regressed on predicted demographic and social variables, followed by perceptions of Housing Satisfaction, Neighborhood Accessibility, Neighborhood Security, and Neighborhood Social Cohesion. Neighborhood variables added significant explanation of variance in both social and emotional loneliness. Mediation tests using PROCESS showed that the effects of all neighborhood variables were mediated by Private-Restricted or Locally Integrated Network types on Social Loneliness only.

**Discussion and Implications:**

These findings highlight the importance of neighborhood factors in relation to feelings of loneliness and the recognition of social network types as mediators of these relationships for social loneliness. The aspects of neighborhoods that prevent loneliness provide directions for planners and prevention programs. Interventions to prevent social loneliness can usefully and practicably focus on the housing and neighborhood environment.

Loneliness is increasingly understood as a public health issue, and the prevalence of loneliness estimated to increase with population aging (Holt-Lunstad, 2017). There is a growing body of evidence for the harmful physical and mental health consequences of loneliness among older people, including dementia, coronary heart disease, diabetes, hypertension and metabolic syndrome, poorer general physical health, functional decline, suicidality, depression, and excess mortality (Cacioppo et al., 2010; Holwerda et al., 2016).

Loneliness has been defined as an emotional response to “a discrepancy between desired and achieved levels of social contact” (Robinson et al., 2013, p. 250). Influential theorizing by Weiss (1973) suggests that there are two different experiences of loneliness: feeling bereft of desired intimacy in close relationships (such as marriage or kinship) or feeling the lack of a broader social network (such as friends and neighbors). These aspects are known and assessed as “emotional loneliness” and “social loneliness” (De Jong Gierveld & Van Tilberg, 2006; Russell et al., 1984) and are important considerations for intervention (Holwerda et al., 2016).

## Predictors of Loneliness

Although people may experience loneliness at any stage of life, older people are seen as vulnerable owing to the higher likelihood of loss of partners, reduced social networks, and restrictions on mobility (McLaughlin et al., 2011; Perkins et al., 2012; Savikko et al., 2005). However, not all old people are lonely, and a range of studies have highlighted who is most at risk for loneliness. A review of such predictors of loneliness in cross-sectional studies (Cohen-Mansfield et al., 2016) found that older adults who experience loneliness were also significantly more likely to be female, unmarried, and older; have lower incomes, educational levels, quality of social relationships, self-reported health, and physical functioning; and to live alone. Psychological health issues associated with loneliness included poorer mental health, negative life events, and cognitive deficits. A systematic review of research in New Zealand (Wright-St Clair et al., 2017) also showed that loneliness was significantly related to being female, Māori (indigenous people of Aotearoa/New Zealand), socially isolated, living alone, and having a visual impairment, depression, or suicidal ideation.

## Interventions to Ameliorate Loneliness

In response to concerns about growing levels of loneliness in many Western societies, a range of interventions has been developed, generally aimed at changing individual behavior and connecting lonely people with others. The most common approaches to intervention include education about personal relationships (Martina et al., 2018); counseling and cognitive training to change maladaptive social cognitions (Masi et al., 2011); befriending programs that typically involve matching individuals with a visitor (Bantry-White et al., 2018); or social group interventions (Hand et al., 2021).

There is little evidence supporting the efficacy of these approaches to intervention (Kharicha et al., 2017). Although there is some weak support for the efficacy of group activities (and very little for one-to-one support and information) these have very limited effectiveness (Dickens et al., 2011; Pynnönen et al., 2018) and are very resource-intensive (Cohen-Mansfield et al., 2016). A more recent umbrella review (Jarvis et al., 2020) reported weak support for any interventions for loneliness.

Qualitative studies provide some insight into older people’s resistance to such interventions. Kharicha et al. (2017) reported that people assessed as lonely did not favor community-based services or one-to-one support such as befriending. Support aimed at loneliness per se discouraged engagement, while social activities based on shared group interests were preferred. Bantry-White et al. (2018) noted that befriending services, while often valued by participants, do not reflect broader community relationships and structures and are therefore less likely to be sustained. Furthermore, such interventions do not reach all lonely people (Park et al., 2019). A review of underrepresented groups among service-users (Moriarty & Manthorpe, 2017) noted that diversity is rarely reported in befriending research. Accordingly, despite many services and activities, the prevalence of loneliness in community-dwelling older people has remained constant (Kharicha et al., 2017). In a review of qualitative studies, Cohen-Mansfield et al. (2016) report that participants raised different issues to those focused on in quantitative surveys. They mentioned environmental barriers, unsafe neighborhoods, migration, housing, and resources for socializing as issues related to loneliness. Social and personal assistance may continue to provide support, but to prevent loneliness we need to consider the broader social and material environment and its contribution to the experience of loneliness among older people.

## Neighborhoods and Loneliness

Berkman and colleagues (2000) proposed an influential public health model that describes a pathway for the effects of the wider environment on health. The model describes how environmental contexts structure social networks, which in turn affect personal responses, including loneliness. Drawing on social theory, Berkman et al. (2000) suggest that structural arrangements in societies shape the resources available to individuals, including their social networks, which provide social resources. The structure of social networks themselves determines individual behaviors and attitudes by shaping the flow of those resources, thus determining opportunities and constraints on behavior. Social networks may be formed based on neighborhood, kinship, friendship, institutional affiliation, or other characteristics. An important contribution of this model has been to highlight the importance of the environmental location of well-being. More recently, there has been increasing recognition of the importance of place and local neighborhoods as settings for the development of social networks or for the everyday experiences of loneliness.

Recent research has demonstrated that the quality of the immediate living environment plays a significant role in shaping older people’s social participation and quality of life (Tomaszewski, 2013). In particular, people’s perceptions of the quality of their neighborhoods have been strongly related to reports of loneliness. People’s perceptions of the social environment of their own neighborhood have also been shown to significantly increase their sense of belonging and lower feelings of exclusion in the United Kingdom (Prattley et al., 2020).

It is theoretically unsurprising that the social environment is strongly related to loneliness; however, perceptions of the physical and service aspects of environments are interrelated with the social environment (Stephens et al., 2019). In Singapore, Wee et al. (2019) found that renting and living in a physical environment perceived as poor was related to loneliness among urban apartment dwellers. More poorly serviced and maintained physical surroundings may influence social interaction and promote loneliness. Victor and Scharf (2005) note that loneliness was related to areas characterized by social deprivation in the United Kingdom and Netherlands. They also speculated that characteristics of more poorly served neighborhoods, such as fear of crime and low levels of trust, might reduce neighborhood friendships.

The Berkman et al. (2000) model suggests that social networks mediate these associations of the environmental context and loneliness, and there is some empirical support for this model. For example, Dykstra and De Jong Gierveld (1999) found that socioeconomic status (SES) was only indirectly related to loneliness; the relationship between higher incomes and lower levels of loneliness was mediated by social network size. In regard to neighborhoods, Scharf and De Jong Gierveld (2008) found that characteristics of social networks were related to both neighborhood perceptions and the experience of loneliness. The interrelationships between different aspects of neighborhoods (physical qualities, facilities, and social cohesion), the environmental formation of supportive social networks, and experiences of loneliness require ongoing enquiry, and the Berkman et al. model provides a framework for mediation hypotheses.

## The Present Study

There are consistent associations between older people’s perceptions of their neighborhood’s characteristics and experiences of loneliness. Neighborhoods provide a clear site for effective intervention; however, more information is required about the nature of the elements of neighborhood environments that could be targets for change.

The present study aimed to assess the relationship of four aspects of the perceived neighborhood environment (satisfaction, security, accessibility, and social cohesion) with emotional and social loneliness and the mediating effects of social networks, while controlling for known predictors of loneliness. We made the following predictions:

(1) Previously identified variables, SES, social network types, social participation, ethnicity, marital status, gender, age, and physical health, would be associated with emotional and social loneliness.
(2) Perceptions of neighborhood qualities would explain additional variance in social and emotional loneliness.
(3) The relationship between perceptions of neighborhood qualities and loneliness would be mediated by social network types.

## Method

### Sample and Design

A cross-sectional study was conducted in Aotearoa/New Zealand. An anonymous questionnaire, with postage-paid return envelope, was distributed via post to 2300 (23.1% Māori) over 55-year-olds living in four Wards (administrative areas) on the Kapiti Coast in 2019. Equal probability random sampling procedures were used to select two independent samples to represent the general population and the Māori population (indigenous people of Aotearoa). Māori was oversampled for this study using the Māori descent indicator on the general electoral roll to maximize participant recruitment. Data were collected between May 22, 2019 and October 7, 2019.

A total of 917 participants (53.2% female [55% in the total population], 16.1% Māori [0.03% in the total population], and 39.9% response rate) completed the questionnaire. The average age of the participants was 75 years (standard deviation [*SD*] = 6.87; range = 61–100 years). Ethnic identity was recorded as: Māori, *N* = 136; Pasifika, *N* = 19; New Zealand European or Pākeha, *N* = 720; Asian, *N* = 6; and Other, *N* = 55. In regard to Marital Status: 577 were in a married, civil union, or de facto relationship; 130 widowed; and 112 living singly.

### Measures

#### Loneliness

The short-form version of the De Jong Gierveld Loneliness Scale (De Jong Gierveld & Van Tilburg, 2006) was administered. The six-item scale includes three items to assess emotional aspects of loneliness (e.g., “I experience a general sense of emptiness”) and three items to assess social (e.g., “There are enough people I feel close to”) aspects of loneliness on a 3-point scale (“no”, “more or less,” and “yes”). Using an item response model scale scores are based on dichotomous item scores, with the answer “more or less” always indicating loneliness. Summing the neutral and positive answers (“more or less” and “yes”) on negatively formulated items provides an emotional loneliness score ranging from 0 to 3 (*M* = 0.53, *SD* = 0.84, α = 0.66, in the present sample). Summing neutral and negative answers (“no” and “more or less”) on the positive items provides a social loneliness score ranging from 0 to 3 (*M* = 1.06, *SD* = 1.16, α = 0.79). Loneliness scale scores are not computed when item scores were missing (De Jong Gierveld & Van Tilburg, 2010).

#### Social networks

Type of social network was assessed using the practitioner assessment of network type instrument (PANT; Wenger & Tucker, 2002). The PANT consists of eight items measuring three domains of social networks: distance, interactions, and engagement which are scored according to a protocol (Wenger, 1997) providing separate scores (ranging from 0 to 13) on five Social Network Types (in which higher scores mean more connections associated with this type of network): A “Family Dependent” support network focused on close family ties with few neighborhood and friend links (*M* = 4.61; *SD* = 1.80); a “Locally Integrated” network including close relationships with local family, friends, and neighbors (*M* = 4.38; *SD* = 2.29); a “Local Self-Contained” network with primary reliance on neighbors (*M* = 5.84; *SD* = 2.06); a “Wider Community” focused network with a high salience of friends (*M* = 6.40; *SD* = 2.26); and a “Private Restricted” network, which has no relatives, few nearby friends, and low levels of community involvement (*M* = 4.58; *SD* = 2.32). The reliability and construct validity of the PANT has been supported among older adults in Aotearoa New Zealand (Szabo et al., 2018).

#### Neighborhood variables

##### Neighborhood social cohesion

The Neighborhood Social Cohesion Tool (Stafford et al., 2003) differentiates among four factors that may influence the quality of interactions in the neighborhood: trust (e.g., most people in this area can be trusted; six items), attachment to the neighborhood (e.g., I really feel part of this area; four items), practical help (e.g., I feel comfortable asking my neighbor to lend me $5; three items), and tolerance or respect (e.g., people in this area treat each other with respect; six items). Items are rated on a 5-point scale anchored at 1 = strongly disagree and 5 = strongly agree. Scores were summed to provide a composite index of neighborhood cohesion. Range in this sample was 75–90 (*M* = 74.06; *SD* = 10.80; α = 0.88).

Neighborhood accessibility was assessed using three items (I can get to the shops easily; I am close to any help I need; I am close enough to important facilities) rated on a 5-point scale anchored at 1 = no, definitely not to 5 = yes, definitely. Scores were summed to provide a range of 3–15 (*M* = 13.75; *SD* = 2.23; α = 0.88).

Housing satisfaction was assessed with eight items (e.g., my house supports all my daily activities; my house is difficult for me to maintain) which were rated on a 5-point scale anchored at 1 = no, definitely not to 5 = yes, definitely. Negative items were reversed and scores summed to provide a range of 14–40 (*M* = 36.02; *SD* = 4.32; α = 0.70).

Neighborhood security was assessed with four items (I feel safe at home; I feel safe in my neighborhood; the neighborhood is peaceful; I have peace of mind at home) which were rated on a 5-point scale anchored at 1 = no, definitely not to 5 = yes, definitely. Scores were summed to provide a range of 8–20 (*M* = 19.25; *SD* = 1.65; α = 0.81).

Higher scores on all neighborhood variables mean more positive perceptions of neighborhood qualities.

#### Social and demographic variables

SES was assessed using a measure of economic living standards for older people (whose income or educational status is not a reliable indicator of SES). The short-form version of the Living Standards Capabilities for Elders (LSCAPE-6; Breheny et al., 2013) consists of six items measuring the capability of health care access, social integration, social contribution, enjoyment of daily activities, sense of security, and autonomy on a 5-point scale anchored at 1 = not at all true for me and 5 = definitely true for me. Range in this sample was 6–30 (*M* = 23.57; *SD* = 5.26; α = 0.78)

Health was assessed with a single item (In general, would you say that your health is …) with responses provided on a 5-point scale ranging from 1 (poor) to 5 (excellent) (*M* = 2.52; *SD* = 0.98).

Social participation was assessed in terms of a social group membership. Participants indicated their membership in a sports club, community or service organizations that help people, political party, professional association, or business organization, trade union, religious, church, or other spiritual organization, a hobby, leisure time, or arts association/group, or group that support cultural traditions, or arts, or other. Positive responses to these items were summed, providing a total group membership score ranging from 0 to 8 (*M* = 2.00; *SD* = 1.39).

Dummy variables were created to assess gender (0 male, 1 female); ethnicity collapsed to two categories (1 Māori, 2 Non-Māori); and marital status (1 partnered [married or de facto], 2 single [never-married, widowed, or separated]).

### Data Analysis

To test H1 and H2 Pearson’s *r* correlation coefficients were calculated. Social or Emotional Loneliness were regressed in two Hierarchical Multiple Regression equations on all variables significantly related to the dependent variables. At step 1 the first set of hypothesized variables was entered, and at step 2 all neighborhood variables were entered to test their additional contribution.

To test H3 we used Haye’s process macro for mediation analysis (Hayes, 2018) to calculate the significance and indirect effect sizes of the proposed mediating relationships.

Mean scores were imputed for those variables that met the criteria of MAR, and less than 20% of items were missing within the variable (Neighborhood Social Cohesion and Housing Satisfaction) without bias (Peyre et al., 2011). Most missing data (140 cases) resulted from items in the Social Network Types measure that were not appropriate for imputation. Using listwise deletion, 212 participants were excluded from the regression equations, but there was no evidence of systematic deletion (*N* = 708).

## Results

### Correlations

Pearson’s *r* bivariate correlation coefficients are shown in Table 1. All Neighborhood variables were significantly negatively correlated with both social and emotional loneliness. Family-Dependent and Private-Restricted social networks were positively related to social loneliness, while Locally Integrated networks were negatively related to social loneliness. Private-Restricted networks were also positively related to emotional loneliness, while Locally Integrated and Wider Community networks were negatively related to emotional loneliness.

**Table 1.** Pearson’s *r* Bivariate Correlation Coefficients Between All Study Variables

Variable	1	2	3	4	5	6	7	8	9	10	11	12	13	14	15	16	17	18
1.Social loneliness	―																	
2.Emotional loneliness	0.349**	―																
3.Family-dependent social network	0.119**	0.058	―															
4.Private-restricted social network	0.344**	0.090*	0.064	―														
5.Local integrated social network	−0.337**	−0.119**	−0.178**	−0.649**	―													
6.Local self-contained network	0.027	0.016	0.126**	−0.227**	−0.384**	―												
7.Wider social network	−0.046	−0.102^**^	−0.043	−0.015	0.034	−0.049	―											
8.Housing satisfaction	−0.321**	−0.402**	0.006	−0.169**	0.162**	0.030	0.146**	―										
9.Neighborhood access	−0.275**	−0.270**	−0.036	−0.150**	0.183**	0.010	0.120**	0.441**	―									
10.Neighborhood security	−0.262**	−0.302**	−0.005	−0.153**	0.134**	0.015	0.074*	0.424**	0.353**	―								
11.Neighborhood social cohesion	−0.378**	−0.240**	−0.217**	−0.346**	0.309**	0.045	0.119**	0.428**	0.345**	0.458**	―							
12.Group belonging	−0.138**	−0.057	0.039	−0.345**	0.323**	−0.150**	0.290**	0.128**	0.138**	0.091**	0.158**	―						
13.Gender	−0.074*	0.095**	−0.007	−0.128**	0.187**	−0.113**	−0.024	−0.071*	−0.049	−0.050	−0.003	0.079*	―					
14.Living standards	−0.250**	−0.295**	−0.016	−0.096**	0.121**	−0.011	0.096**	0.464**	0.327**	0.302**	0.341**	0.148**	−0.079*	―				
15.Health	0.222**	0.247**	0.027	0.106**	−0.088*	−0.014	−0.128**	−0.303**	−0.291**	−0.183**	−0.210**	−0.159**	0.004	−0.318**	―			
16.Age	0.027	0.039	−0.050	0.053	0.015	−0.124**	0.047	−0.027	−0.005	0.124**	0.077*	0.021	−0.024	−0.040	0.207**	―		
17.Marital status	0.081*	0.176**	−0.052	0.009	0.143**	−0.185**	−0.068	−0.093**	−0.108^**^	−0.076*	−0.025	0.017	0.277**	−0.123**	0.071*	0.171**	―	
18.Ethnicity	−0.024	0.030	−0.068	0.097**	−0.094**	−0.011	0.111**	0.053	−0.019	−0.029	0.046	−0.043	−0.032	0.105^**^	−0.046	0.117**	−0.138**	―

*Note:* *p < .05; **p < .01.

Group membership was negatively related only to social loneliness. Of the social and demographic variables, only age and ethnicity were not related to loneliness.

### Regression Analyses

#### Social loneliness as the dependent variable

The results of this regression equation are shown in Table 2. Of the variables entered in Step 1, Living Standards, Health, and belonging to a Locally Integrated Social Network were negatively related to Social Loneliness. Males and Single people were also more likely to report Social Loneliness. Family-Dependent and Private-Restricted Social Networks were positively related to Social Loneliness. Together these variables explained 22% of the variance in Social Loneliness with Living Standards and Social Networks as the strongest predictors.

**Table 2.** Hierarchical Regression of Social Loneliness on All Demographic, Social, and Neighborhood Predictors (*N* = 708)

	Step 1		Step 2	
Variable	β	*t*	β	*t*
Demographic/social variables				
Gender	−0.08*	−2.31	−0.10^**^	−3.04
Marital status	0.10**	2.75	0.10**	2.90
Living standards	−0.19***	−5.26	−0.07*	−1.84
Health	−0.11**	2.96	0.05	1.44
Group membership	−0.03	−0.75	−0.02	−0.53
Family social network	0.10**	2.76	0.07*	2.15
Private social network	0.18**	2.90	0.17**	2.75
Local integrated social network	−0.17**	−2.70	−0.10	−1.62
Local self-contained network	−0.00	−0.06	0.04	0.80
Wider social network	0.01	0.34	0.04	1.17
Neighborhood variables				
Housing satisfaction			−0.13**	−3.21
Neighborhood accessibility			−0.09*	−2.48
Neighborhood security			−0.04	−1.06
Neighborhood social cohesion			−0.15**	−3.47
*R* ^2^	0.229		0.292***	
Adj. *R*^2^	0.218		0.277***	
*R* ^2^ Change			0.062***	

*Note:* *p < .05; **p < .01; ***p < .001.

At Step 2, the Neighborhood variables explained an additional 6% of variance. Housing Satisfaction, Neighborhood Accessibility, and Neighborhood Social Cohesion were negatively related to Social Loneliness after accounting for the social and demographic predictors.

#### Emotional loneliness as the dependent variable

The results of this regression equation are shown in Table 3. Of the variables entered in Step 1, Living Standards were negatively related to Emotional Loneliness. Single people were more likely to report Emotional Loneliness, and Health was positively related to Emotional Loneliness. Together these variables explained 15% of the variance in Emotional Loneliness with Marital Status as the strongest predictor.

**Table 3.** Hierarchical Regression of Emotional Loneliness on All Demographic, Social and Neighborhood Predictors (*N* = 708)

	Step 1		Step 2	
Variable	β	*t*	β	*t*
Demographic/social variables				
Gender	0.04	1.15	0.01	0.35
Marital status	0.14***	3.65	0.13***	3.78
Living standards	−0.25***	−6.51	−0.11**	−2.79
Health	0.14***	3.67	0.08*	2.08
Group membership	0.00	0.05	0.00	0.05
Family social network	0.04	1.04	0.04	1.18
Private social network	−0.00	−0.04	−0.00	−0.04
Local integrated social network	−0.08	−1.20	−0.02	−0.31
Local self-contained network	0.01	0.10	0.04	0.77
Wider social network	−0.05	−1.36	−0.03	−0.70
Neighborhood variables				
Housing satisfaction			−0.20***	−4.78
Neighborhood accessibility			−0.08	−1.94
Neighborhood security			−0.14**	−3.50
Neighborhood social cohesion			−0.02	−0.50
*R* ^2^	0.161***		0.242***	
Adj. *R*^2^	0.149***		0.227***	
*R* ^2^ Change			0.081***	

*Note:* *p < .05; **p < .01; ***p < .001.

At Step 2, the Neighborhood variables explained an additional 8% of variance. Housing Satisfaction, and Neighborhood Security were negatively related to Emotional Loneliness after accounting for the social and demographic predictors.

### Mediation

To test H3 a simple mediation analysis was performed for each predicted pathway from neighborhood factors, through social network types, to Social or Emotional Loneliness (the outcome variables). Predictor variables were Neighborhood Security, Neighborhood Accessibility, Neighborhood Social Cohesion, and Housing Satisfaction. The mediator variables for each pathway were those related to the outcome variable. For Social Loneliness, these were Private, Family, and Locally Integrated Social Networks. For Emotional Loneliness, the mediators tested were Private, Wider, and Locally Integrated Social Networks.

The relationship between Neighborhood Security and Social Loneliness was mediated by Private (indirect effect = −0.0207, 95% confidence interval [CI; −0.0356, −0.0083]) and Locally Integrated Social Network scores (indirect effect = −0.0154, 95% CI [−0.0278, −0.0059]).

The relationship between Neighborhood Accessibility and Social Loneliness was mediated by Private (indirect effect = −0.0168, 95% CI [−0.0285, −0.0076]) and Locally Integrated Social Network scores (indirect effect = −0.0146, 95% CI [−0.0246, −0.0059]).

The relationship between Neighborhood Social Cohesion and Social Loneliness was mediated by Private (indirect effect = −0.0061, 95% CI [−0.0098, −0.0025]) and Locally Integrated Social Network scores (indirect effect = −0.0046, 95% CI [−0.0077, −0.0018]).

The relationship between Housing Satisfaction and Social Loneliness was mediated by Private (indirect effect = −0.0074, 95% CI [−0.0138, −0.0027]) and Locally Integrated Social Network scores (indirect effect = −0.0067, 95% CI [−0.0117, −0.0026]).

Family Social Network was not a mediator.

**Figure 1.**
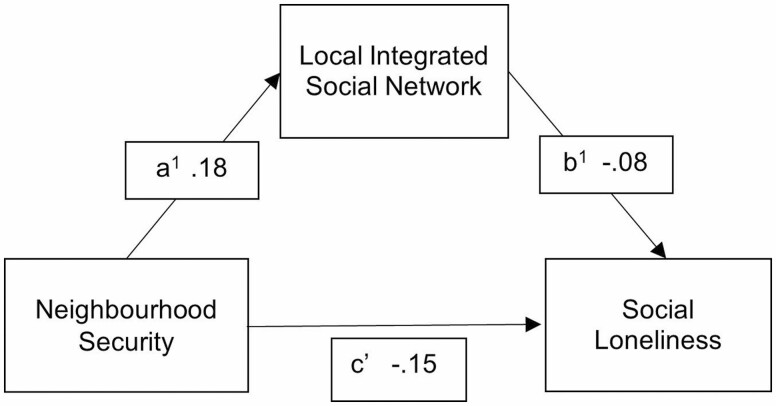
Mediation of the relationship between Neighborhood Security and Social Loneliness by Locally Integrated Social Network scores showing significant pathway coefficients.

The pattern of these relationships is exemplified in Figure 1, and path coefficients for all significant relationships are reported in Table 4.

**Table 4.** Pathway Coefficients for Mediating Relationships Between Neighborhood Factors, Social Network Types, and Social Loneliness

Predictor	Mediator	a^1^ coefficient	b^1^ coefficient	c’ coefficient
Neighborhood security	Private social network	−0.21**	.010**	−0.15**
	Local integrated social network	0.18**	−0.08**	−0.15**
Neighborhood accessibility	Private social network	−0.16**	0.10**	−0.13**
	Local integrated social network	0.20**	−0.07**	−0.13**
Neighborhood social cohesion	Private social network	−0.07**	0.08**	−0.03**
	Local integrated social network	0.06**	−0.07*	−0.03**
Housing satisfaction	Private social network	−0.08**	0.09**	−0.07**
	Local integrated social network	0.08**	−0.08**	−0.07**

*Note:* *p < .01; **p < .001. a1 coefficient, effect of neighbourhood security on locally integrated social network; b1coefficient, effect of local integrated social network on social loneliness; c’ coefficient, effect of neighbourhood security on social loneliness.

There were no significant mediators of the relationship between neighborhood variables and Emotional Loneliness.

### Discussion and Implications

#### Predictors of loneliness

The first main finding is the importance of the neighborhood to people’s feelings of loneliness over and above known social and demographic predictors of loneliness. H1 was partially supported. Being male, single, having lower living standards, poorer health, and belonging to family-dependent and private-restricted network types were related to higher social loneliness. Being single, having better health, lower living standards, and low scores on locally integrated networks were related to higher emotional loneliness. As predicted in H2, neighborhood perceptions, including housing satisfaction, accessibility to important services, sense of security, and neighborhood social cohesion, provided additional explanations for social or emotional loneliness. European studies (Kemperman et al., 2019; Scharf & De Jong-Gierveld, 2008; Shiovitz-Ezra, 2015) have also demonstrated that perceptions of the physical, service, safety, and social aspects of neighborhood quality are related to loneliness among older people.

#### Neighborhoods and social and emotional loneliness

For loneliness prevention, our findings also point to different needs for different aspects of loneliness. It is not surprising that housing satisfaction, social cohesion, and access to facilities were related to the development of the broader social network types, such as those including friends and neighbors, that are negatively related to social loneliness. Research has generally shown that living in a rural setting, having few social contacts, a small network size, and lack of network support are more highly correlated with social rather than emotional loneliness (Dahlberg & McKee, 2014). More recently, Victor and Pikhartova (2020) summarized existing evidence demonstrating the effects of living environments on social well-being. Social loneliness, seen by Weiss (1973) as the absence of a desired social network, may be more amenable to the influence of the neighborhood, as shown by our finding that social networks mediated the relationship between neighborhood factors and social loneliness only.

Dahlberg and McKee (2014) also showed that in a UK sample, social factors explained the most variance in social loneliness, and it is these social factors that may be understood as affected by neighborhood provisions. Bridge (2002, p. 2) noted that “neighborhood” has commonly been defined as “fostering the development of social networks through interaction in local public space.” Although the importance of the provisions of neighborhoods for social network formation has been debated, there is limited research into the effects of neighborhood provisions on the development of local social networks (Wissink & Hazelzet, 2012). These authors showed that, in Japan, the neighborhood does play a role in the development of social networks for older people (compared to other groups); however, more research in this area is needed to support new community development policies.

As in our study, Dahlberg and McKee (2014) also found that psychological and health factors explained most of the emotional loneliness. The focus of emotional loneliness on the need for intimate attachment can also explain the importance of housing satisfaction and neighborhood security found here, rather than the social aspects of the neighborhood. Although these findings together suggest that social loneliness may be best addressed through neighborhood-level intervention, while individually focused intervention may be more helpful for alleviating emotional loneliness, there has been relatively little research using measures that differentiate between emotional and social loneliness, and almost none examining the effects of the housing or neighborhood environment on social and emotional loneliness. Our findings highlight the importance of recognizing these two different aspects of loneliness, particularly for use in approaches to intervention (Holwerda et al., 2016).

#### Social network types

The findings that family-dependent and private-restricted social networks were positively related to social loneliness, while locally integrated networks were negatively related to social loneliness, draw attention to the specific types of social networks that place older people at greater risk for social loneliness. Kim et al. (2018) showed that older adults with very constrained social networks were more likely to feel lonely. Wenger (1997) noted that those with local family-dependent or private-restricted networks are most at risk for loneliness and other mental illness, whereas those in locally integrated networks are at the least risk. The present findings support this prediction, but only for social loneliness. Those in more private-restricted or family-dependent networks were also more likely to report higher social loneliness, whereas those in more locally integrated networks were less likely to report social loneliness. While marital status was a stronger predictor of emotional loneliness, this type of loneliness was not a feature of any social network type.

#### Social networks mediate the effects of neighborhood on social loneliness

Theorizing these sets of relationships using the Berkman et al. (2000) model strengthens our understanding of the present findings and provides pointers to particular aspects of neighborhoods to be targeted. Only the relationships between neighborhoods and social loneliness were mediated by social network types. This is comprehensible in that the wider environment can affect the broader social relationships that prevent social loneliness, whereas it is more likely to be intimate relationships (such as with a spouse) that affect emotional loneliness.

The results of mediation testing highlight positive and negative pathways to social loneliness. The positive pathway, to lower social loneliness, is provided by locally integrated networks that are supported by higher perceptions of housing satisfaction, accessibility, security, and social cohesion in the neighborhood. The negative pathway is through private-restricted networks, which are related to poorer perceptions of all neighborhood factors. Although family restricted networks were related to social loneliness, they are not influenced by the neighborhood features. This can be understood in terms of the essentially more individual and lifetime composition of these types of networks (Antonucci et al., 2013).

The Berkman et al. (2000) model describes social networks as mediating structures between broad environmental pressures such as industrialization and urbanization and individual responses; however, it does not provide explanations of the specific effects of neighborhoods on social networks and loneliness. Researchers such as Tomaszewski (2013) have provided empirical support for the marked effects of living in disadvantaged areas on social participation, frequency of contact, and access to social support. While contributing to the growing evidence base, such authors provide little additional explanation for these effects apart from the common understanding that neighborhoods may be particularly important in shaping older people’s social networks because of the greater amount of time spent in the home. As the evidence base for the importance of neighborhoods grows, more research is needed to investigate the specific ways in which neighborhood qualities affect the development of supportive social networks.

#### Limitations

Our measure of social participation (membership in social organizations and groups) was surprisingly unrelated to any aspect of loneliness, and this may be a measurement issue. Improved measures of actual social participation should be explored. Because SES is an important explanatory variable, its role beyond the control variable should be explored. People of lower SES are also more likely to live in less well-serviced neighborhoods (e.g., Scharf & De Jong Gierveld, 2008), and these inequalities should be taken into account. Interactions of neighborhood variables with SES could be included in future modeling. Some of our participants pointed out that we neglected to ask whether people were living in retirement villages, which are a growing feature of living arrangements for older people. These, or other particular types of environments, should be taken into account when assessing neighborhood characteristics. Another limitation that must be considered is the cross-sectional nature of this study. The theoretically derived hypotheses suggest one direction of effects; however, it is equally possible that loneliness and social network type affect people’s perceptions of their neighborhoods.

#### Implications

These findings have important implications for the prevention of loneliness among older people. In particular, this study highlights that interventions to prevent social loneliness can usefully and practicably focus on the housing and neighborhood environment. Such interventions are feasible at policy and regulatory levels and include town planning for types of housing or inclusion of intergenerational groups, provision of facilities that support social interaction such as parks, libraries, and local shops, and safe environments for movement (World Health Organization, 2007). Many of the previous intervention efforts that have not proved successful (Jarvis et al., 2020) have been largely aimed at encouraging social participation among individuals, while the broader social structures that provide natural opportunities for interaction (Bantry-White et al., 2018) are neglected. Neighborhood qualities are aspects of the environment that may be influenced by central and local government policy and planning. Aspects such as housing design, provision of footpaths and lighting, transport, libraries, shops, and services (Alidoust & Bosman, 2015), and development of neighborhood social cohesion (Scharf & De Jong-Gierveld, 2008; Wickes et al., 2019) may be provided for by social policy, intervention, and regulation.

A second important highlight is the different needs of those suffering from emotional loneliness rather than social loneliness. Individual factors such as SES, health, or single marital status that contribute to emotional loneliness will require focused individual intervention support and services. And these may be developed with the recognition of the nature of emotional loneliness and the need for intimate connection.

This study highlights the importance of neighborhoods in relation to feelings of loneliness, the importance of social network types as mediators of the relationships between these neighborhood factors and social loneliness, and the various aspects of neighborhoods related to social or emotional loneliness that provide directions for planners and prevention programs. Future studies could usefully provide further evidence to develop this explanatory model of neighborhoods and loneliness.
